# Diagnosis of SARS-CoV-2 Infection with LamPORE, a High-Throughput Platform Combining Loop-Mediated Isothermal Amplification and Nanopore Sequencing

**DOI:** 10.1128/JCM.03271-20

**Published:** 2021-05-19

**Authors:** Leon Peto, Gillian Rodger, Daniel P. Carter, Karen L. Osman, Mehmet Yavuz, Katie Johnson, Mohammad Raza, Matthew D. Parker, Matthew D. Wyles, Monique Andersson, Anita Justice, Alison Vaughan, Sarah Hoosdally, Nicole Stoesser, Philippa C. Matthews, David W. Eyre, Timothy E. A. Peto, Miles W. Carroll, Thushan I. de Silva, Derrick W. Crook, Cariad M. Evans, Steven T. Pullan

**Affiliations:** a Oxford University Hospitals NHS Foundation Trust, Oxford, United Kingdom; b Nuffield Department of Medicine, University of Oxford, Oxford, United Kingdom; c Public Health England, National Infection Service, Salisbury, United Kingdom; d Sheffield Teaching Hospitals NHS Foundation Trust, Sheffield, United Kingdom; e Sheffield Biomedical Research Centre, Sheffield Bioinformatics Core, University of Sheffield, Sheffield, United Kingdom; f Big Data Institute, Nuffield Department of Population Health, University of Oxford, Oxford, United Kingdom; g Centre for Tropical Medicine and Global Health, Nuffield Department of Medicine, University of Oxford, Oxford, United Kingdom; h The Florey Institute for Host-Pathogen Interactions, Department of Infection, Immunity and Cardiovascular Disease, University of Sheffield, Sheffield, United Kingdom; i NIHR Health Protection Research Unit in Healthcare Associated Infections and Antimicrobial Resistance, University of Oxford in partnership with Public Health England, Oxford, United Kingdom; Cepheid

**Keywords:** diagnosis, LamPORE, nanopore sequencing, SARS-CoV-2

## Abstract

LamPORE is a novel diagnostic platform for the detection of severe acute respiratory syndrome coronavirus 2 (SARS-CoV-2) RNA combining loop-mediated isothermal amplification with nanopore sequencing, which could potentially be used to analyze thousands of samples per day on a single instrument. We evaluated the performance of LamPORE against reverse transcriptase PCR (RT-PCR) using RNA extracted from spiked respiratory samples and stored nose and throat swabs collected at two UK hospitals. The limit of detection of LamPORE was 10 genome copies/μl of extracted RNA, which is above the limit achievable by RT-PCR, but was not associated with a significant reduction of sensitivity in clinical samples. Positive clinical specimens came mostly from patients with acute symptomatic infection, and among them, LamPORE had a diagnostic sensitivity of 99.1% (226/228; 95% confidence interval [CI], 96.9% to 99.9%). Among negative clinical specimens, including 153 with other respiratory pathogens detected, LamPORE had a diagnostic specificity of 99.6% (278/279; 98.0% to 100.0%). Overall, 1.4% (7/514; 0.5% to 2.9%) of samples produced an indeterminate result on first testing, and repeat LamPORE testing on the same RNA extract had a reproducibility of 96.8% (478/494; 94.8% to 98.1%). LamPORE has a similar performance as RT-PCR for the diagnosis of SARS-CoV-2 infection in symptomatic patients and offers a promising approach to high-throughput testing.

## INTRODUCTION

Rapid, reliable, high-throughput methods of testing for severe acute respiratory syndrome coronavirus 2 (SARS-CoV-2) infection would help to control transmission. Present diagnosis relies mostly on reverse transcriptase PCR (RT-PCR), but it has proven difficult to expand to the scale needed for population-wide testing of symptomatic individuals. For example, shortages of laboratory RT-PCR capacity still limit the United Kingdom testing program over a year after the SARS-CoV-2 pandemic was declared a public health emergency of international concern by the WHO. Further expansion of testing to include screening of asymptomatic individuals, which may be needed to prevent SARS-CoV-2 circulation, would require a significant further increase in testing capacity (1, 2).

In the United Kingdom, clinical laboratories have struggled to expand conventional RT-PCR workflows to meet the demand for SARS-CoV-2 testing, and many have explored alternative methods that would be more scalable or allow near-patient use (3, 4). At the Oxford University Hospitals NHS Foundation Trust (OUH) and Sheffield Teaching Hospitals NHS Foundation Trust (STH), we evaluated LamPORE, a novel diagnostic platform for SARS-CoV-2 developed by Oxford Nanopore Technologies (ONT) that combines loop-mediated isothermal amplification (LAMP) with nanopore sequencing (5). During sample preparation, a unique combination of DNA barcodes are incorporated into the LAMP products from each specimen so that they can be pooled into a single sequencing run. In the current protocol, up to 92 samples can be analyzed on 1 flow cell, potentially allowing thousands of samples to be analyzed per day on a single instrument running multiple flow cells in parallel. The workflow involves a 40-minute amplification, followed by library preparation, and a 60-minute sequencing run, generating results in a comparable time to RT-PCR when starting with extracted RNA.

As well as molecular barcoding, using sequencing to detect the outcome of the LAMP reaction offers other advantages compared with simpler LAMP assays that detect the presence of DNA synthesis by measurement of pH, turbidity, or fluorescent dyes. Sequenced reads from a specific target will contain sequences not present in the primers, avoiding false positives caused by nonspecific amplification (although the amplicons are not large enough to usefully genotype the virus) (6). Conversely, reads confidently assigned to SARS-CoV-2 targets may indicate a true positive even if present at relatively low levels, potentially improving the low sensitivity seen in several LAMP assays compared with RT-PCR (7, 8). In addition, the detection of LAMP products by sequencing allows the possibility of multiplexing the assay with other pathogens. LamPORE uses ONT flow cells compatible with several sequencing instruments, including the portable MinION device and high-throughput GridION and PromethION platforms, and thus, it could potentially be used both for mobile and centralized testing.

In this evaluation, we compare the performance of LamPORE with RT-PCR on extracted RNA from respiratory specimens. Initially, we use spiked samples to determine the analytical limit of detection of the assay. We then use stored clinical samples to determine the assay’s diagnostic sensitivity, specificity, and reproducibility.

## MATERIALS AND METHODS

The evaluation was conducted across three sites, namely, OUH, STH, and the Public Health England National Infection Service at Porton Down (PHE Porton Down).

### LamPORE.

LamPORE is a CE marked diagnostic assay developed by ONT and described in detail in James, et al. (5). The assay was performed identically at each site using a GridION instrument with operators unaware of reference PCR results. It takes 20-μl RNA input into a single multiplex reaction targeting the following three regions of the SARS-CoV-2 genome using previously published primers (9): ORF1a, envelope, and nucleocapsid genes, plus human β-actin mRNA as a control of sampling adequacy and assay performance. LamPORE sample preparation uses a 96-well plate format, with each sample having 1 of 8 LAMP forward inner primer (FIP) barcodes and 1 of 12 transposase (rapid) barcodes added before pooling. In these experiments, a single LAMP barcode (FIP7) was not used, as it had previously been associated with lower β-actin read counts and was awaiting replacement (unpublished data). As a result, plates contained 80 samples, plus 2 no-template controls and 2 positive controls consisting of synthetic SARS-CoV-2 RNA (Twist Bioscience). To assess for potential sample-sample contamination, positive and negative clinical samples were intermixed, with positions altered between replicates.

We used the LamPORE protocol dated 1 July 2020 (version 1, revision 4). Briefly, this protocol consists of adding sample RNA to LAMP master mix and primers and then incubating the mixture at 65°C to 80°C in a thermocycler for 40 minutes, during which time amplification occurs and the LAMP primer barcodes are incorporated into concatemers containing the target sequence. Following these steps, samples from the same column are pooled and a second set of barcodes are incorporated using a rapid transposase-based method. All samples are then pooled into a single sequencing library, with no need for normalization, as DNA concentrations are similar in all positive samples following LAMP, regardless of the initial viral load. The pooled library has a magnetic bead cleanup, then is added to a MinION flow cell, and sequenced for 60 minutes, after which a report is generated automatically by the instrument within seconds for each barcode set. Unlike RT-PCR, LamPORE does not provide the equivalent of a cycle threshold (*C_T_*) value reflecting the initial viral load, as measurement occurs only after amplification is complete. The number of reads assigned to each target is used to generate a report as follows: (i) invalid, <50 classified reads in total detected from SARS-CoV-2 and β-actin targets, (ii) positive, ≥50 SARS-CoV-2 reads detected (adding read counts across all three SARS-CoV-2 targets), (iii) Inconclusive, not invalid and ≥20 and <50 SARS-CoV-2 reads detected, and (iv) negative, not invalid and <20 SARS-CoV-2 reads detected.

### Spiked samples—PHE Porton Down.

Spiked samples were prepared and analyzed at PHE Porton Down to establish the limits of detection of LamPORE. Aliquots of pooled volunteer saliva were used for spiking experiments, which were confirmed SARS-CoV-2 negative by RT-PCR. They were spiked with cultured SARS-CoV-2 (Victoria/01/202026 passaged twice in Vero/hSLAM cells) at 1,000 SARS-CoV-2 genome copies/ml of sample and serially diluted with the remaining material to create a dilution series of positive samples.

RNA was extracted from 360 μl of the spiked sample using the QiaAMP viral RNA minikit (Qiagen), with RNA eluted in 36 μl. Reference RT-PCR was conducted with the CDC NS1 assay with 5-μl RNA input (10). Quantification was determined by comparison to a standard curve of a plasmid 2019-nCoV_N positive control (Integrated DNA Technologies). Further details are in the supplemental material.

### Clinical specimens—OUH and STH.

Testing of stored clinical samples was performed at OUH and STH. All samples were nose and/or throat swabs collected into viral transport media during routine clinical care and stored at −80°C.

### (i) Sample selection.

*(a) SARS-CoV-2-positive samples.* At OUH, sequentially available positive specimens collected from March to April 2020 were chosen without reference to the RT-PCR cycle threshold (*C_T_*) value. During this time, a uniplex RdRp RT-PCR assay was in use, based on the assay described by Corman et al. (11). At STH, a stratified random sample of specimens collected from April to May 2020 were selected based on their initial SARS-CoV-2 E gene *C_T_* value, using an in-house assay based on the Corman et al. protocol (11, 12), with 50% chosen to have *C_T_* values of <30 and 50% to have ≥30. At both sites, testing was largely restricted to hospitalized patients and symptomatic staff during the collection period.

*(b) SARS-CoV-2-negative samples.* At OUH, negative samples were selected from stored prepandemic respiratory samples. They had initially been tested with either GeneXpert Flu/RSV (Cepheid) or the BioFire FilmArray respiratory panel 2.0 (bioMérieux) and were purposefully chosen to include samples with a range of other respiratory pathogens. Over 90% of samples were collected between October and December 2019, but those samples containing non-SARS-CoV-2 seasonal coronaviruses were used up until a collection date of 10 March 2020 to increase the number available. At STH, negative samples were selected from among those submitted for SARS-CoV-2 testing.

### (ii) RNA extraction.

For samples originating from OUH, RNA extraction was conducted with the QIAsymphony SP instrument and the DSP virus/pathogen kit (Qiagen) (13). A total of 200 μl of viral transport medium was extracted, and RNA was eluted in 60 μl. For samples originating from STH, RNA extraction was performed using the MagNA Pure 96 instrument with the MagNA Pure 96 DNA and viral neuraminidase (NA) small volume kit (Roche). A total of 200 μl of viral transport medium was extracted, and RNA was eluted in 100 μl. Aliquots of RNA were stored at −80°C prior to analysis.

### (iii) Reference RT-PCR.

Reference RT-PCR was undertaken contemporaneously with LamPORE on aliquots of the same RNA extract, with operators unaware of LamPORE results. For samples originating from OUH, the reference RT-PCR was the RealStar SARS-CoV-2 RT-PCR assay (Altona Diagnostics) using 10-μl RNA input. For samples originating from STH, an in-house RT-PCR assay based on Corman et al. methods was used with 6-μl RNA input (11, 12). Further details are in the supplemental material.

### (iv) Replicates.

To assess the reproducibility of the assay, LamPORE replicates were performed on aliquots of the same RNA extract. To ensure comparable RT-PCR and LamPORE results between OUH and STH, a subset of samples was exchanged between sites, with LamPORE and reference RT-PCR repeated.

### Statistical analysis.

R version 3.5.0 was used for analysis with exact binomial confidence intervals calculated for proportions. Initial LamPORE replicates were used to derive estimates of sensitivity and specificity, with second replicates used to estimate LamPORE reproducibility. Results are reported in line with the Standards for Reporting Diagnostic accuracy studies (a STARD checklist is in the supplemental material).

### Ethics.

The process for collection of the donated saliva was approved by the PHE Research Ethics and Governance Group. The protocol for the use of stored clinical samples at OUH and STH was reviewed by the Institutional Review Board of OUH and the University of Oxford, and it was determined that the activity constituted service evaluation and service development. As such, it did not need research ethics review.

## RESULTS

### Limit of Detection.

Using samples spiked with cultured SARS-CoV-2, LamPORE had a limit of detection of 1,000 SARS-CoV-2 genome copies/ml of sample and detected 15/15 samples (Table 1). With the RNA extraction protocol used, and assuming 100% extraction efficiency, this limit of detection would correspond to a concentration of 10 genome copies/μl of extracted RNA (or 200 copies per 20-μl reaction). Although LamPORE did not consistently detect spiked samples at concentrations below this value, it was positive in 8/18 (44%) samples at a concentration of 100 copies/ml of sample, corresponding to 1 genome copy/μl of extracted RNA (or 20 copies per 20-μl reaction). By comparison, RT-PCR using the CDC NS1 assay was also positive in 15/15 samples at 1,000 SARS-CoV-2 genome copies/ml of sample and in 14/18 (78%) of samples at 100 copies/ml of sample, although the difference with LamPORE was not statistically significant (*P* = 0.09 by Fisher’s exact test).

**TABLE 1:** Limit of detection of LamPORE using spiked samples^*a*^

	SARS-CoV-2 genome copies^*b*^				LamPORE results (*n*)			RT-PCR results	
No. of replicates	Per ml sample	Per 20-μl LamPORE reaction	Per 5-μl RT-PCR reaction	Per μl of extracted RNA	Positive	Negative	Inconclusive/invalid	RT-PCR positive (*n*)	Mean *C_T_* value ± SD
15	1,000	200	50	10	15	0	0	15/15	32.1 ± 0.5
18	100	20	5	1	8	10	0	14/18	35.7 ± 1.0
18	10	2	0.5	0.1	0	18	0	0/18	ND^*c*^
15	0	0	0	0	0	15	0	0/15	ND

a Copies/RT-PCR is calculated for the comparator CDC NS1 RT-PCR assay using 5-μl RNA input volume.

b The relationship between copies/ml sample and copies/μl extracted RNA applies to the extraction method used here, in which RNA from a 360-μl sample was eluted in 36 μl.

c ND, not determined.

### Diagnostic performance.

Diagnostic performance of LamPORE was assessed using 514 stored nose and throat swabs, 400 from OUH and 114 from STH (details in Table S1 in the supplemental material). Requesting location was available for 135/150 (90%) SARS-CoV-2-positive samples from OUH but not for other samples. Among these samples, 41 (30%) were from outpatient locations (including occupational health), 24 (18%) were from community hospitals, 45 (33%) were from emergency departments or acute admission wards, and 25 (19%) were from other inpatient locations. Sixty cross-site replicates demonstrated good correlation between RT-PCR *C_T_* values for E gene targets at OUH and STH despite different assays being used, so this was used as the reference *C_T_* (see Fig. S1 in the supplemental material). Samples were analyzed on a total of 13 LamPORE runs performed on separate days.

Among 229 RT-PCR-positive samples tested by LamPORE, 226 were reported positive and 2 were reported negative, giving an overall diagnostic sensitivity of 99.1% (226/228; 95% CI, 96.9% to 99.9%) (Table 2). All valid samples at *C_T_* values of 34.9 or lower were positive by LamPORE (Table 3). Considering performance at lower viral loads, 7/9 samples with a *C_T_* value of ≥35 were positive and 22/22 of those with *C_T_* values between 30 and 34.9 were positive. Both false-negative samples by LamPORE had *C_T_* values of ≥38, and 1 of them was positive by LamPORE on repeat testing (see Table S2 in the supplemental material). The one RT-PCR-positive sample that was invalid on initial LamPORE testing was correctly positive when repeated.

**TABLE 2:** Clinical diagnostic performance of LamPORE vs RT-PCR

RT-PCR result	LamPORE result (*n*)				
	Positive	Negative	Inconclusive	Invalid	Total
Positive	226	2	0	1	229
Negative	1	278	3	3	285
Total	227	280	3	4	514

**TABLE 3:** Performance of LamPORE in the SARS-CoV-2-positive clinical samples by RT-PCR E gene *C_T_* value

RT-PCR *C_T_* value	LamPORE result (*n*)				
	Total	Positive	Negative	Inconclusive	Invalid
<15	23	23	0	0	0
15.0–19.9	51	51	0	0	0
20.0–24.9	73	72	0	0	1
25.0–29.9	51	51	0	0	0
30.0–34.9	22	22	0	0	0
>35	9	7	2	0	0

**FIG 1:**
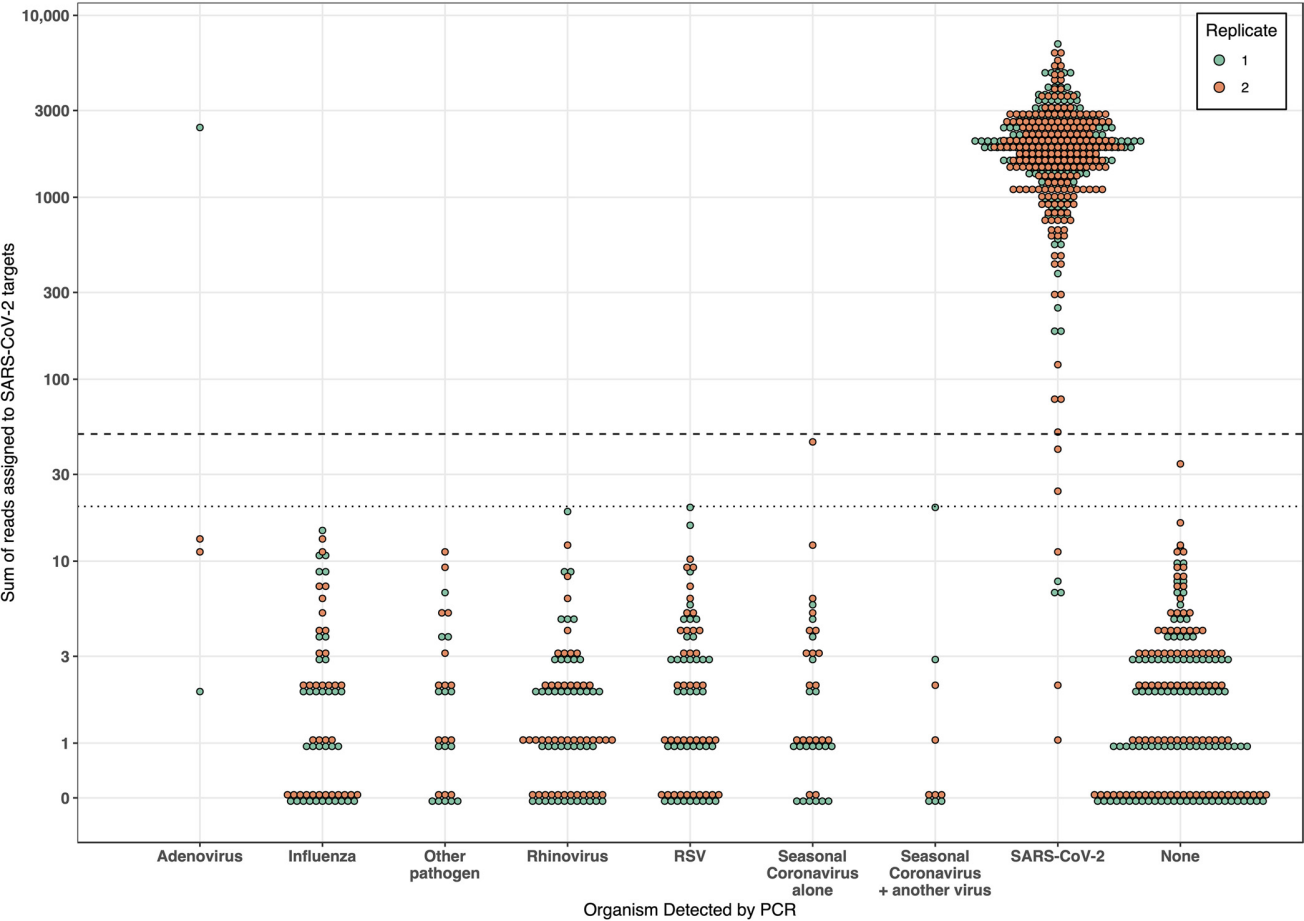
Analytical specificity of LamPORE in samples positive for a range of respiratory pathogens. Data for both LamPORE replicates are shown. The dashed line is the threshold for a positive result (≥50 reads), and the dotted line is the threshold for an inconclusive result (≥20 reads). “Other pathogens” includes parainfluenza virus (*n* = 10), Mycoplasma pneumoniae (*n* = 3), and human metapneumovirus (*n* = 1). Invalid samples are plotted.

Of 285 RT-PCR-negative samples, 278 were negative and 1 was positive by LamPORE, giving an overall diagnostic specificity of 99.6% (278/279; 98.0% to 100.0%) (Table 2). The false positive was a prepandemic respiratory sample that was also positive for adenovirus and which had 2,419 SARS-CoV-2 reads detected. However, this sample was negative on repeat LamPORE testing (Fig. 1). Six RT-PCR-negative samples gave indeterminate results (three invalid, three inconclusive), of which four were correctly negative on repeat testing, one remained invalid, and one was not retested. Overall, among both RT-PCR-positive and -negative samples, 1.4% (7/514; 0.5% to 2.9%) produced an indeterminate result on first testing.

Another respiratory pathogen was detected by multiplex RT-PCR in 153 negative samples, including 43 with rhinovirus, 38 with respiratory syncytial virus (RSV), 33 with influenza, and 24 with seasonal coronaviruses (9 HKU1, 7 NL63, 7 OC43, and 1 229E). Overall, there was no evidence that the presence of any other respiratory pathogen was associated with false-positive results or greater numbers of reads assigned to SARS-CoV-2 targets (Fig. 1).

**FIG 2:**
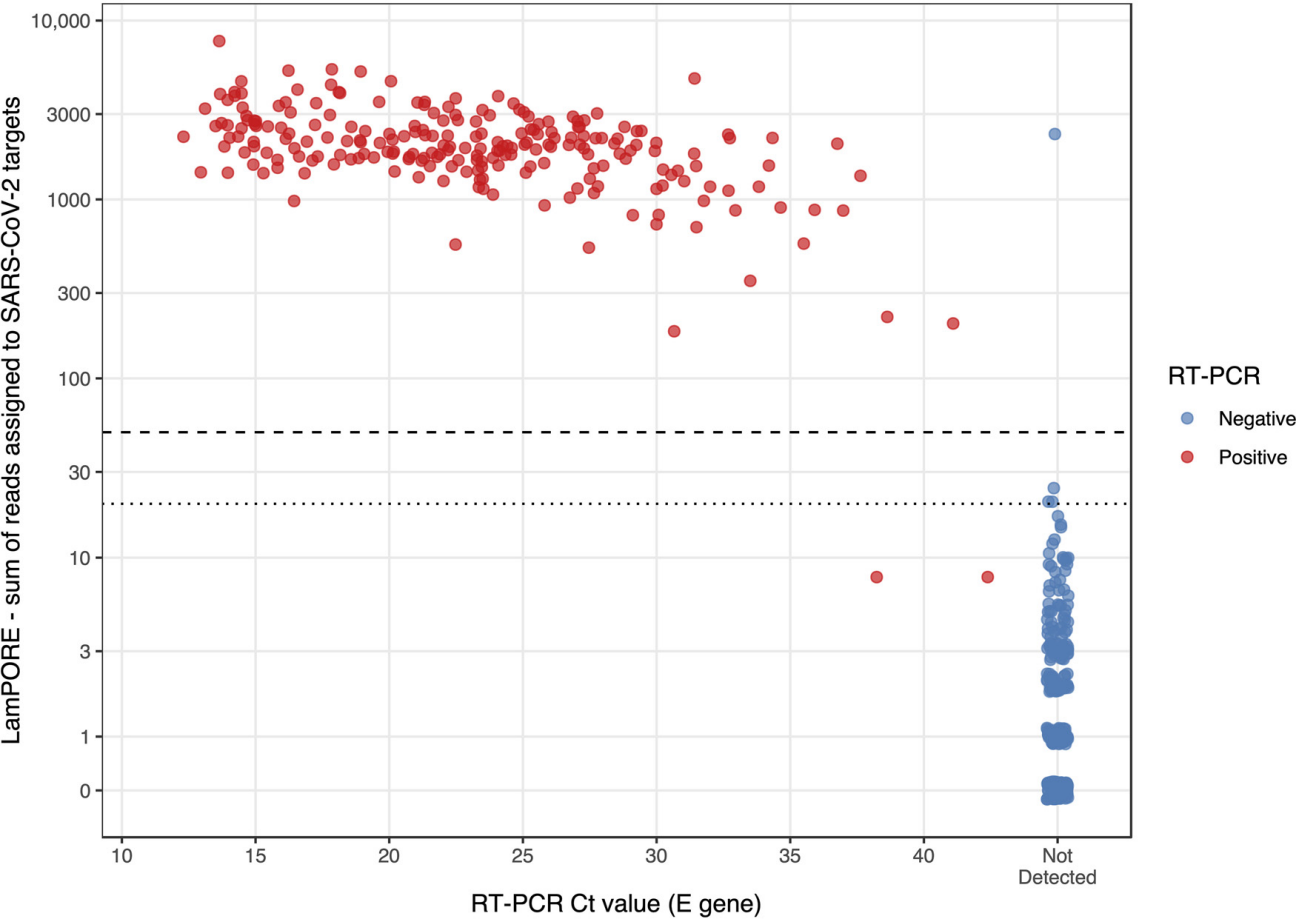
Total number of reads assigned to SARS-CoV-2 targets by LamPORE versus RT-PCR E gene *C_T_* value. The dashed line is the threshold for a positive result (≥50 reads), and the dotted line is the threshold for an inconclusive result (≥20 reads). Results are for replicate 1 only. Invalid samples are not shown.

As well as the categorical result produced by the LamPORE reporting algorithm, RT-PCR results were compared with the number of reads assigned by LamPORE to SARS-CoV-2 targets (Fig. 2). This comparison showed that the prespecified cutoff of ≥50 for a positive result was optimal, with any cutoff in the range of 25 to 182 producing a maximal Youden index (sensitivity + specificity − 1) of 0.988. As the rate at which reads are detected becomes roughly constant after a few minutes of sequencing, the effect of a sequencing run longer or shorter than 60 min can be inferred. All samples reported positive by LamPORE had >180 SARS-CoV-2 reads detected and so would have been positive after 30 min of sequencing, at which point there would have been no increase in indeterminate results. Conversely, extending the sequencing duration with the same diagnostic thresholds would not have allowed the detection of either of the two false-negative samples without producing large numbers of false positives.

### Reproducibility.

LamPORE was repeated on 494 samples using the same RNA extract and produced identical results in 478, giving an overall reproducibility of 96.8% (478/494; 94.8% to 98.1%) (Table S3 and S4 in the supplemental material). In four samples (0.8%) with discrepant LamPORE results, the same sample switched between negative and positive. In the other 12 discrepant samples, LamPORE replicates included 1 indeterminate result. All 90 cross-site LamPORE replicates performed between Oxford and Sheffield were concordant (60 RT-PCR/LamPORE positive and 30 RT-PCR/LamPORE-negative). Combining results from replicates to assess the rate of possible sample-sample contamination, 1/576 (0.2%; 0.0% to 1.0%) negative samples or no-template controls were positive by LamPORE.

## DISCUSSION

LamPORE has been identified by the UK government as a possible high-throughput platform that could alleviate shortages in SARS-CoV-2 testing capacity (14). In a manuscript released by its developers, LamPORE correctly detected SARS-CoV-2 in 79 of 80 clinical specimens (98.8%; 95% CI, 93.2% to 100.0%), although no SARS-CoV-2-negative specimens were available for testing. Instead, the assay was tested on 85 nonrespiratory human RNA extracts, and 4 were incorrectly reported as positive (sensitivity, 95.2%; 88.3% to 98.7%), which the authors attributed to probable sample contamination (5). In this evaluation, we found that LamPORE had a high diagnostic sensitivity (99.1%) and specificity (99.6%) in our clinical sample set. Combined with a high reproducibility (96.8%) both within and across sites, these results support its practical use for high-throughput testing in a low-prevalence population. Although the assay we evaluated targeted SARS-CoV-2 alone, the LamPORE platform could be adapted to detect other pathogens and is amenable to multiplexing in order to target multiple pathogens in the same assay.

The limit of detection of LamPORE, at 10 genome copies/μl of extracted RNA, was somewhat higher than the 2 copies/μl achievable in previous evaluations of high-performance RT-PCR (15), but this value did not correspond to a significant loss of diagnostic sensitivity in the clinical samples. In our spiking experiments, RNA was extracted from 360 μl transport medium and eluted in 36 μl, a 10-fold concentration. This amount is higher than that of most commonly used extraction protocols, for example, those used at OUH and STH produced 3-fold and 2-fold concentrations, respectively. Therefore, the limit of detection, measured in genome copies/ml of sample, using LamPORE with a high-concentration extraction would be similar to PCR as commonly used with a low-concentration extraction. Automated, commercially available extraction methods can produce a 20-fold RNA concentration, which could further improve the limit of detection, although higher degrees of concentration could lead to assay inhibition, so this would need further evaluation.

Although no clinical metadata were available about the individuals whose samples were used in this evaluation, they would have mainly been derived from patients with acute symptomatic infection, often requiring admission to hospital, as testing was mainly limited to this group during the first wave of infection. The distribution of *C_T_* values may be higher in a population with more mild or asymptomatic infections and would be markedly higher among those who remain RT-PCR positive weeks after recovering from acute infection (16). Our data suggest that LamPORE is most likely to miss weakly positive samples with *C_T_* values above 35 and thus could have had lower diagnostic sensitivity if tested in such groups. However, this may not be a significant practical disadvantage, as although weak positives have some value for contact tracing, they are likely to come from individuals with low infectious potential (17, 18).

Our evaluation has several limitations. It was conducted after the first wave of COVID-19 in the United Kingdom, when there were few incident cases, so we were unable to prospectively collect samples and instead relied on frozen transport media, which could differ from fresh material. Sample collection occurred at a time when there was little genetic variation in SARS-CoV-2, and we did not attempt to assess the possible effect of future sequence variation causing failure in any of the three gene targets. Positives were defined by a positive RT-PCR at the time of initial sample collection and by repeat positive RT-PCR simultaneously with LamPORE, but although RT-PCR is used as a reference test for SARS-CoV-2, there are many reports of its suboptimal sensitivity in clinical infection (19).

This early evaluation of LamPORE compared its performance against RT-PCR using extracted RNA, as this is the standard material used for the detection of SARS-CoV-2. However, the requirement for viral inactivation and RNA extraction and the additional need for LamPORE library preparation could lead to bottlenecks that would mitigate the potential benefit of LamPORE for high-throughput or mobile testing. LAMP reactions are reported to be more robust than RT-PCR to inhibitors present in clinical samples and so may have superior performance with extraction-free protocols (20, 21). The use of such extraction-free protocols could greatly streamline the workflow, but further evaluation is required. We also did not evaluate how the throughput and turnaround time of LamPORE would compare with RT-PCR during routine use in a clinical laboratory or centralized testing center. The benchtop GridION instrument can accommodate five flow cells simultaneously and so could analyze over 3,000 samples in a 12-hour day at two-thirds occupancy, and the PromethION instrument has a theoretical capacity more than 10-fold higher, but using LamPORE to test tens or hundreds of thousands of samples per day would be dependent on a streamlined workflow, including automated sample handling, integration with laboratory information management systems, and careful safeguards to minimize the risk of contamination.

In conclusion, we show that LamPORE on extracted RNA offers a promising method of high-throughput SARS-CoV-2 testing. However, further evaluation in mild or asymptomatic infection is needed, and large-scale use requires the development of streamlined workflows, possibly by including simpler sample preparation to avoid the need for conventional RNA extraction.
